# Effects of Non-Invasive Ventilation Combined with Oxygen Supplementation on Exercise Performance in COPD Patients with Static Lung Hyperinflation and Exercise-Induced Oxygen Desaturation: A Single Blind, Randomized Cross-Over Trial

**DOI:** 10.3390/jcm8112012

**Published:** 2019-11-18

**Authors:** Maud Koopman, Martijn A. Spruit, Frits M.E. Franssen, Jeannet Delbressine, Emiel F.M. Wouters, Denny Mathew, Anton Vink, Lowie E.G.W. Vanfleteren

**Affiliations:** 1 Department of Research & Development, CIRO, Center of Expertise for Chronic Organ Failure, 6085 NM Horn, The Netherlands; martijnspruit@ciro-horn.nl (M.A.S.); jeannetdelbressine@ciro-horn.nl (J.D.);; 2 NUTRIM, School of Nutrition and Translational Research in Metabolism, 6200 MD Maastricht, The Netherlands; 3 Department of Respiratory Medicine, Maastricht University Medical Center (MUMC), 6202 AZ Maastricht, The Netherlands; 4 REVAL—Rehabilitation Research Center, BIOMED—Biomedical Research Institute, Faculty of Rehabilitation Sciences, Hasselt University, 3590 Diepenbeek, Belgium; 5 Philips Research, 5656 AE Eindhoven, The Netherlands; anton.vink@philips.com (D.M.); denny.mathew@philips.com (A.V.); 6 COPD Center, Sahlgrenska University Hospital, Institute of Medicine, University of Gothenburg, SE-413 45 Gothenburg, Sweden

**Keywords:** Non-invasive ventilation, COPD, static hyperinflation, exercise tolerance

## Abstract

The effects of non-invasive ventilation (NIV) in addition to supplemental oxygen on exercise performance in patients with chronic obstructive pulmonary disease (COPD) with hyperinflation and exercise-induced desaturation (EID) remain unclear. We hypothesized that these patients would benefit from NIV and that this effect would be an add-on to oxygen therapy. Thirteen COPD patients with a residual volume >150% of predicted, normal resting arterial oxygen pressure (P_a_O_2_) and carbon-dioxide pressure (P_a_CO_2_) and EID during a six-minute walk test were included. Patients performed four constant work-rate treadmill tests, each consisting of two exercise bouts with a recovery period in between, wearing an oronasal mask connected to a ventilator and oxygen supply. The ventilator was set to the following settings in fixed order with clockwise rotation: Sham (continuous positive airway pressure (CPAP) 2 cm H_2_O, FiO_2_ 21%), oxygen (CPAP 2 cm H_2_O, FiO_2_ 35%), NIV and oxygen (inspiratory positive airway pressure (IPAP) 14 cm H_2_O/expiratory positive airway pressure (EPAP) 6 cm H_2_O, inspired oxygen fraction (FiO_2_) 35%), intermittent (walking: Sham setting, recovery: NIV and oxygen setting). During the first exercise, bout patients walked further with the oxygen setting compared to the sham setting (225 ± 107 vs 120 ± 50 meters, *p* < 0.05), but even further with the oxygen/NIV setting (283 ± 128 meters; *p* < 0.05). Recovery time between two exercise bouts was shortest with NIV and oxygen. COPD patients with severe static hyperinflation and EID benefit significantly from NIV in addition to oxygen during exercise and recovery.

## 1. Introduction

Ventilatory inefficiency and exercise-induced oxygen desaturation (EID) are key determinants of exercise intolerance in patients with severe COPD [1,2]. Ventilatory inefficiency represents an abnormal increase in the ratio of minute ventilation to carbon dioxide production [3], and combined with expiratory flow limitation causes dynamic hyperinflation [4,5]. Ventilatory inefficiency is related to greater mechanical constraints, worsening pulmonary gas exchange, higher dyspnea scores and poorer exercise capacity [6]. Excess breathing at higher lung volumes has negative hemodynamic consequences resulting in impaired oxygen delivery and/or utilization by the skeletal muscles [7].

EID can also be a result of a ventilation/perfusion mismatch during exercise, caused by diffusion impairment and loss of elastic recoil of the lungs, which is associated with emphysema [8]. The combination of these pathophysiologic features, which occurs in about 25% of the COPD patients [9], results in exertional dyspnea and early exercise termination [10].

Particularly COPD patients with chronic hypercapnic respiratory failure respond well to non-invasive ventilation (NIV) during exercise. Glöckl et al. recently showed that high inspiratory pressure NIV improved cycle endurance time, reduced exercise-induced hypercapnia and reduced exertional dyspnea [11]. In addition, NIV during exercise training in patients with chronic hypercapnic respiratory failure and long term NIV and long-term oxygen supplementation resulted in improvement of cycling endurance time after training [12].

To date, the effects of NIV in addition to supplemental oxygen during exercise and recovery from exercise in non-hypercapnic COPD patients with lung hyperinflation and EID are less studied. NIV can reduce the load of overburdened inspiratory muscles and reduce the work of breathing. The use of inspiratory pressure support during exercise increases endurance time and reduces dyspnea in patients with COPD [13,14]. Unloading respiratory muscles by proportional assisted ventilation in COPD patients improves leg muscle oxygenation, dyspnea and leg fatigue symptoms and prevents exercise-induced diaphragmatic fatigue [15,16]. Moreover, a preliminary study suggested that bi-level positive airway pressure (BiPAP) during recovery after treadmill walking can reduce recovery time [17]. Oxygen supplementation during exercise can improve endurance time, stabilize oxygen saturation and reduce dyspnea in COPD patients [18,19], mainly in those with resting hypoxemia or EID [20]. Improved exercise-capacity with oxygen has shown to be, at least partly, explained by a reduction in DH related to a decreased breathing frequency for a given exercise [21]. The combination of NIV and oxygen during ramp-incremental cycle ergometry in COPD patients with EID and hyperinflation can improve central hemodynamics during exercise; however, this study did not show an improvement of peak work rate when using NIV and oxygen [22].

We hypothesized that non-hypercapnic patients with COPD with lung hyperinflation and EID would benefit from NIV and that this effect would be an add-on to oxygen therapy. Consequently, we aimed to study the effects of NIV in addition to oxygen therapy, compared to oxygen alone, or no support on exercise duration, perceived dyspnea and time to recovery from exercise in this disease phenotype.

## 2. Methods

### 2.1. Patients

Clinically stable COPD patients confirmed by post-bronchodilator spirometry [23], aged 40–80 years, with a residual volume >150% of predicted, resting arterial oxygen pressure (P_a_O_2_) > 7.3 kPa, resting arterial carbon-dioxide pressure (P_a_CO_2_) < 6.5 kPa, and transcutaneous oxygen saturation (SpO_2_) during a 6-minute walking test (6MWT) < 88% were included. Patients were excluded if they had an active malignancy, previous pulmonary surgery, unstable cardiovascular disease, orthopedic problems that could impair the walking tests, and/or if they had to use a walking aid or wheelchair. Lastly, patients were not included when they had a contraindication for the use of BiPAP (i.e., acute sinusitis, otitis media, low blood pressure, inability to adequately clear secretions) as determined by the chest physician. Patients were recruited during a baseline assessment for an inpatient comprehensive pulmonary rehabilitation (PR) program at CIRO (Horn, the Netherlands) between January and December 2016. The study-related tests were performed during the first two weeks of PR.

The study protocol was approved by the medical ethical committee of Maastricht University Medical Center, the Netherlands, filed under NL54204.100.15 R15.077/VOS COPD. Informed consent was signed by all patients.

### 2.2. Sample Size

We performed a sample size calculation using GPower 3.1.9 (Heinrich Heine Universität Düsseldorf, Germany), based on the hypothesis that NIV during recovery will result in a clinically relevant improvement of recovery time of at least 40 seconds, according to a study with 19 patients who used NIV during recovery after treadmill walking compared to a baseline control test [17]. For the sample size calculation, we used a mean of 126 seconds, standard deviation 13 seconds, significance level α = 0.05 and power 1-β = 0.80. We used an effect size of 0.40 and an estimated moderate correlation between the repeated measurements of 0.5, which provided a sample size of 12. Considering a 10% drop-out rate, we estimated an allocation sample size of 13 patients.

### 2.3. Baseline Assessment

Patients underwent a series of tests and questionnaires as part of a routine three-day pre-rehabilitation assessment [24]. The chest physician performed a physical examination and reviewed the patient’s medical history. Post-bronchodilator forced expiratory volume in the first second (FEV1) and forced vital capacity (FVC) were collected using standardized spirometry (MasterScreen^®^ Body, Carefusion, Germany). Additionally, whole-body plethysmography was performed to assess residual volume (RV) and carbon monoxide transfer factor (DLCO, by single breath-hold method) was determined (MasterScreen^®^ Body, Carefusion, Germany). Prior to lung function measurement, 200 μg of salbutamol was provided via an autohaler device. All measurements, executed by a trained lung function technician, were repeated at least three times to ensure repeatability [25]. Reference values for spirometry and lung volume measurements were in accordance with the official statement of the European Respiratory Society [26,27]. Among other tests, patients also performed two 6MWTs. The test with the longest 6-minute walk distance (6MWD) was used for further analysis and to define the treadmill walking speed [28].

### 2.4. Interventions

**Figure 1:**
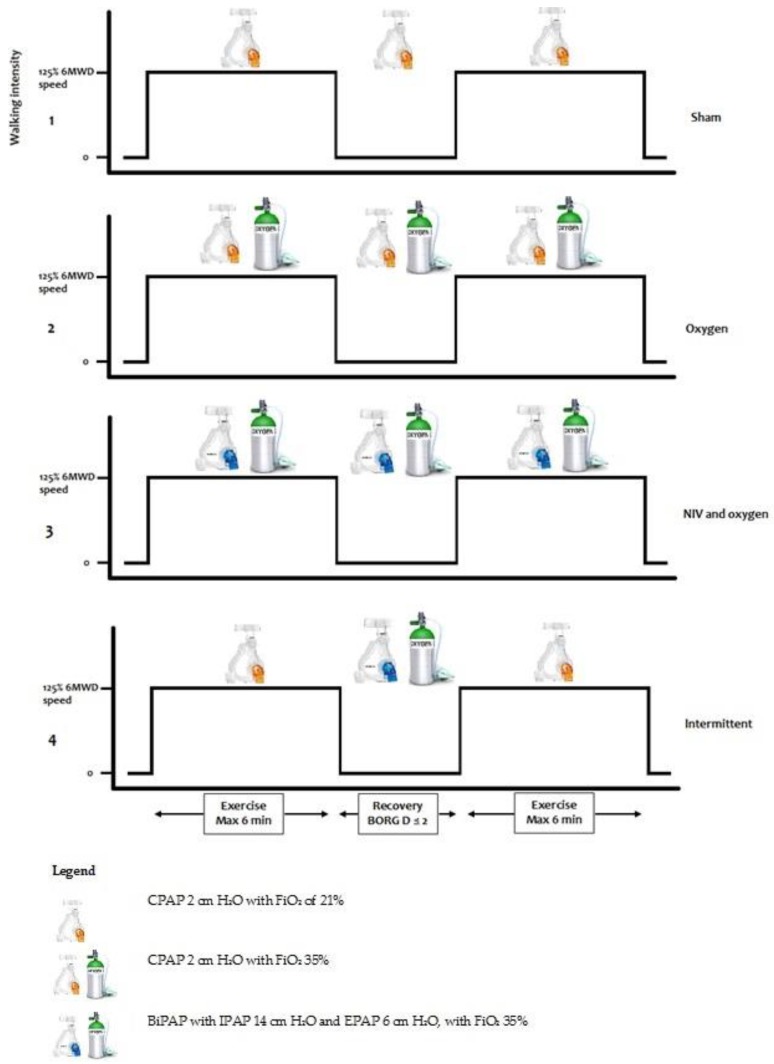
Interventions. Abbreviations: 6MWD = six-minute walking distance, NIV = non-invasive ventilation, BORG D = Borg dyspnea, CPAP = continuous positive airway pressure, BiPAP = bilevel positive airway pressure, FiO_2_ = inspired oxygen fraction.

An overview and description of the different exercise settings are presented in Figure 1. Each test consisted of two bouts of exercise with a timed recovery period in between. The following tests were performed on two consecutive days, with one set of tests in the morning, and one set in the afternoon on both days:

(1) Setting 1 (‘Sham’): Continuous positive airway pressure (CPAP) 2 cm H_2_O with an inspired oxygen fraction (FiO_2_) of 21%.
(2) Setting 2 (‘Oxygen’): CPAP 2 cm H_2_O with FiO_2_ 35%.
(3) Setting 3 (‘NIV and oxygen’): BiPAP with inspiratory positive airway pressure of 14 cm H_2_O and expiratory positive airway pressure of 6 cm H_2_O, with FiO_2_ 35%.
(4) Setting 4 (‘Intermittent’): Setting 1 during walking (2 cm H_2_O), when the patient had an unintended stop the setting was switched to setting 3 (IPAP 14, EPAP 6 cm H_2_O). When the patient started walking again, the setting was switched back to setting 1.

The CPAP pressures were chosen to overcome the pressure gradients of the tube and mask, and were the lowest available pressures on the device. The BiPAP pressures were based on the pressures used when NIV is introduced in the acute clinical setting. The intermittent setting was included to specifically identify whether the use of NIV and oxygen helps shorten recovery time following a bout of exercise.

Randomization of tests was done with a fixed order of tests, but the clockwise rotation of the starting test for each consecutive patient. All patients were blinded for the settings.

To measure recovery time, i.e., the time they needed to recover enough to restart exercise, patients were asked to perform two consecutive constant work rate treadmill walking tests with a timed recovery period in between. Before the start of the first exercise bout, patients were instructed to walk as long as possible, with a maximum duration of six minutes. With the completion of the first exercise bout patients stayed on the treadmill and were instructed to start with the second exercise bout the moment they recovered to a Borg dyspnea score ≤2. Patients were then again instructed to walk as long as possible, with a maximum duration of six minutes. The start of their second bout of exercise was marked as the end of the recovery period. The walking speed at the treadmill was set at 125% of the mean walking speed during the 6MWT. This speed was chosen to increase the likelihood that the patient will stop walking within six minutes.

The sham and NIV support and oxygen supply during the treadmill tests were provided by the Philips Respironics V60 ventilator connected to a wall oxygen supply, using an oronasal mask connected by a tube to the ventilator.

All tests were supervised by a physician who watched over the settings of the non-invasive ventilator and the oxygen supplementation. A biomedical engineer instructed the patients and guided all exercise tests. During testing days, patients did not perform any endurance training. They were allowed to perform resistance training if they felt able to do so.

Treadmill walking time and distance, Borg dyspnea (D) and fatigue (F) score, transcutaneous PaCO_2_ (tcPaCO_2_) and SpO_2_, heart rate (HR), breathing frequency (BF) and tidal volume (Vt) were collected throughout the tests. The V-Sign System from Sentec was used to measure tcP_a_CO_2_, SpO_2_ and HR, with a sensor connected to the earlobe. In two patients, measurements of tcP_a_CO_2_, SpO_2_ and HR with the Sentec device gave an unreliable signal, and SpO_2_ and HR were measured with a Nonin WristOx2 wrist pulse-oximeter. BF and Vt were continuously measured by the V60. All parameters were noted before and after each treadmill test. Because the parameters on the Sentec and the V60 changed every five seconds, all parameters were also noted 10 seconds before and after the start and stop, the mean of these three numbers was finally taken into the calculations. To get continuous data of the parameters, a GoPro HERO action camera was set to film the monitors of the Sentec and V60. The vital data were compared within the four test settings at 20/40/60/80/100% of isotime, in which the shortest walking time was set at 100% isotime. Minute ventilation was calculated by multiplying BF and Vt.

All tests were performed in the same training room, air-conditioned in temperature and humidity, on the same treadmill, and the same ventilator was used in all patients.

### 2.5. Statistical Analysis

Statistics were performed using SPSS version 23.0. Data were analysed for outliers and normality. Results were expressed as mean ± SD when normally distributed, otherwise as median and interquartile range, or count (%). Differences in exercise duration, recovery, Borg D and F, P_a_CO_2_, SpO_2_, HR, BF and Vt were compared using a one-way repeated measures ANOVA. Participants with missing data were excluded from the ANOVA analyses. A generalized estimated equation (GEE) analyses were used with all available data. Statistical significance was assumed if a two-tailed *p*-value was <0.05.

## 3. Results

**Figure 2:**
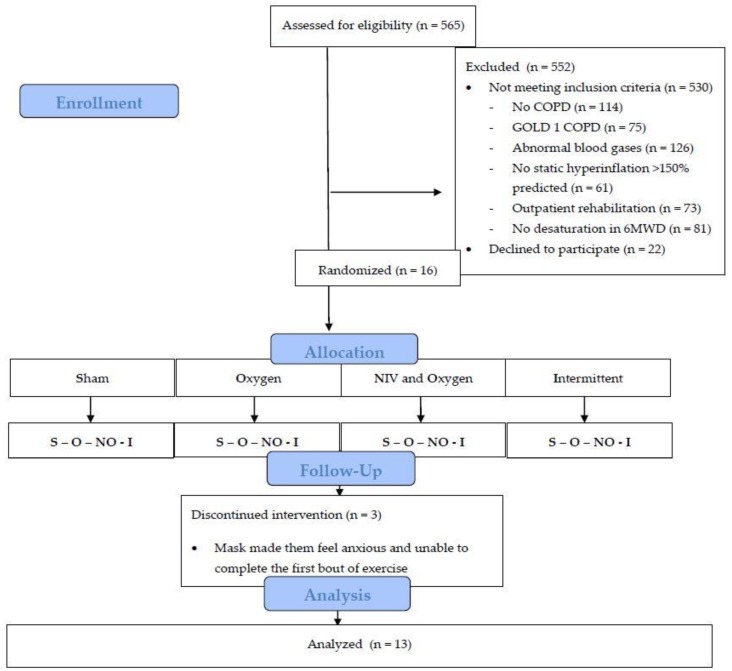
Study flow chart. Abbreviations: COPD = chronic obstructive pulmonary disease, 6MWD = six minute walking distance, NIV = non-invasive ventilation.

Five hundred fifty-two subjects took part in a regular rehabilitation program after an initial assessment. Of the screened patients, 530 did not meet the inclusion criteria, and 22 declined to participate (Figure 2). Sixteen patients volunteered to participate. Thirteen patients completed the four exercise settings. Three patients withdrew informed consent as the mask made them feel anxious and unable to complete the first bout of exercise. Baseline characteristics of the 13 patients (nine women) who completed all four tests are outlined in Table 1. Patients generally had severe airflow obstruction, severe static hyperinflation, and EID during the 6MWD (lowest SpO_2_ during 6MWT: 82.5 ± 5.1%).

### Treadmill Test Outcomes

**Figure 3:**
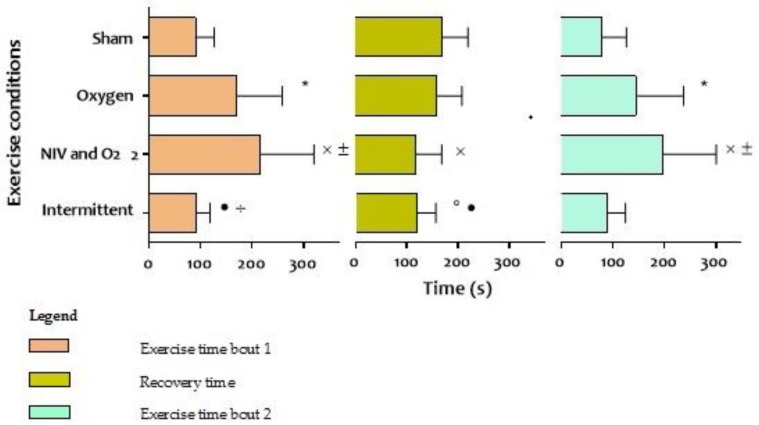
Results of walking time bout 1 and 2 and time till recovery in four tests with different ventilator settings. Statistical significant differences (*p* < 0,05); * = sham vs. oxygen, ˟ = sham vs. NIV and O_2_, ° = sham vs. intermittent, ± = oxygen vs. NIV and O_2_, • = oxygen vs. intermittent, ÷ = NIV and O_2_ vs. intermittent; Abbreviations: NIV = non-invasive ventilation, O_2_ = oxygen, s = seconds.

**Figure 4:**
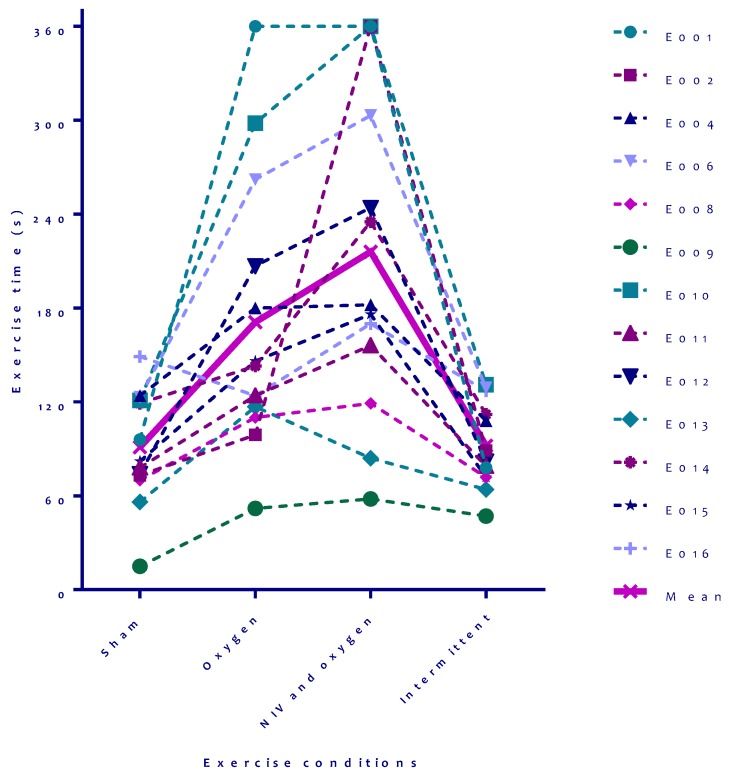
Individual results with different settings on the first bout of exercise. Abbreviations: s = seconds, NIV = non-invasive ventilation.

Compared to sham, patients were able to walk further during a bout of exercise when oxygen was continuously provided (mean 120 ± 50 meters versus 225 ± 107 meters, respectively; *p* = 0.03, Table 2), but recovery time did not significantly change. Total endurance time of exercise bout one and two was longest in the setting with NIV and supplemental oxygen (mean 414 ± 208 seconds, Figure 3), with the shortest recovery time in between two exercise bouts (mean 117 ± 51 seconds, Table 2, Figure 3). Recovery time was comparable when NIV and oxygen were only provided during recovery (setting 4; mean 119 seconds, Table 2, Figure 3). Despite the shortened recovery, patients were not able to walk further after recovery compared to the sham setting. Individual results on the four tests before recovery are shown in Figure 4, individual results after recovery are not shown but were comparable.

Although patients walked furthest with setting 3, no increase in Borg D or F was noted. Alternatively, Borg D was lower after the first test in the oxygen/NIV setting compared to the oxygen setting. Interestingly, leg fatigue was lowest after recovery during the test in which patients walked with sham settings and recovered with NIV and oxygen (Table 2).

No desaturation was seen when walking with oxygen or oxygen/NIV, compared to marked desaturation when walking with setting 1 and 4. Saturation normalized in all test settings during recovery (Table 2).

tcP_a_CO_2_ levels were comparable before starting the first exercise bout (mean tcP_a_CO_2_ 5.1-5.5 kPa, Table 2), but increased at a significantly faster rate when providing oxygen without NIV during the first exercise bout. tcP_a_CO_2_ levels continued to increase during recovery, but stayed levelled during the second round of tests (mean tcP_a_CO_2_ 6.0-6.6 kPa, Table 2). There were no significant differences in tcP_a_CO_2_ levels at the end of the four sets between the four settings.

No significant results between the treadmill tests were found when looking at differences in BF, Vt and ventilation after exercise and recovery (Table 2).

**Figure 5:**
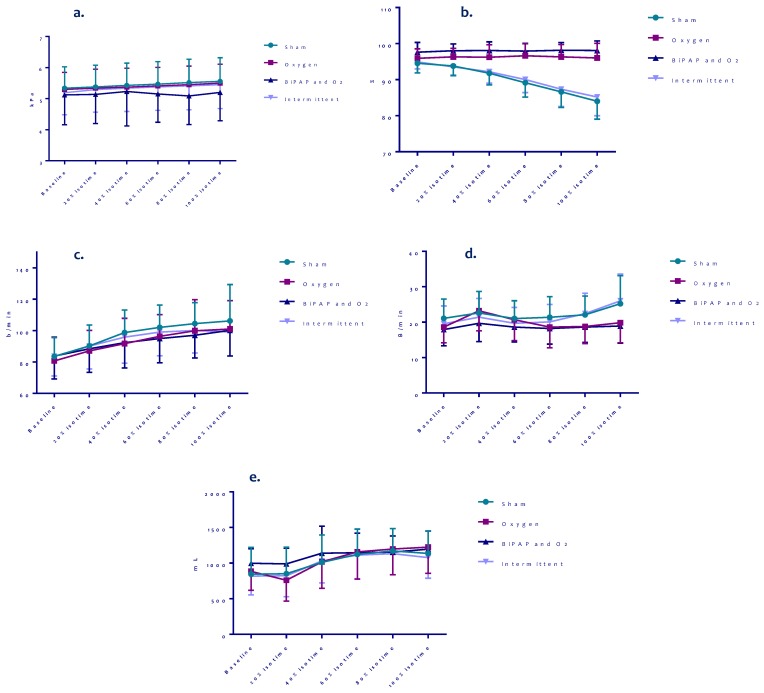
Isotime results on P_a_CO_2_, SpO_2_, heart rate, breathing frequency and tidal volume of different settings during bout 1 of all patients. (**a**) P_a_CO_2_ test 1, (**b**) SpO_2_ test 1, (**c**) Heart rate test 1, (**d**) Breathing frequency test 1, (**e**) Tidal volume test 1.

Figure 5 shows the results of the isotime values on tcP_a_CO_2_, SpO_2_, HR, BF and Vt of the first round of treadmill tests. SpO_2_ declined with a significantly steeper slope with sham or intermittent settings compared to oxygen with or without NIV, but SpO_2_ values between the oxygen and oxygen/NIV setting were comparable. The trends of tcP_a_CO_2_, HR and BF seemed to be lowest, and Vt seemed to be highest during exercise with NIV and oxygen compared to the other settings, but these differences were not significant.

## 4. Discussion

This study showed that normoxemic COPD patients with severe hyperinflation and EID significantly improved exercise endurance time with oxygen supplementation during exercise, which further improved when NIV was added to oxygen. Endurance time increased with the same level of dyspnea and faster post-exercise recovery. The combination of NIV and oxygen supplementation provided only during recovery after exercise shortened recovery time with less leg fatigue, but did not improve subsequent exercise endurance time after recovery.

Oxygen supplementation in COPD patients has previously shown to decrease the respiratory rate and thereby reduce DH in hyperinflated patients [18,19]. We observed no differences in the ventilatory response to exercise and DH was not measured. Correction of desaturation on itself has been shown to improve exercise capacity [18,19], which might be one of the explanatory factors for improved exercise in our patients.

This study shows that NIV, in addition to oxygen therapy, significantly further improves the exercise capacity in non-hypercapnic, severely hyperinflated patients with EID. The physiological mechanism behind this improvement is clearly beyond the correction of EID. A physiological study in COPD patients with EID, using non-invasive open ventilation utilizing a volume-regulation ventilation system, showed that although this type of ventilation does decrease the inspiratory effort as measured by decreasing accessory inspiratory muscle EMG, exercise endurance was not significantly affected by non-invasive ventilation alone if oxygen was not administered [29]. Moreover, use of the non-invasive open ventilation system was associated with a slower breathing rate and a higher oxygen saturation than generally achieved using oxygen, without a change in pulmonary gas exchange efficiency.

It has been described that by providing BiPAP, the inspiratory threshold load to initiate inspiration is lowered, expiration is eased by preventing small airways closure, and DH might be decreased [30]. Acute-on-chronic hyperinflation in COPD patients may result in a reduction in peripheral muscle blood flow, due to the central hemodynamic consequences or cardiac output redistribution towards the overloaded respiratory muscles [31,32]. In addition, decreased ventilatory efficiency and desaturation can contribute to this process.

A previous study explored the effects of additional NIV to oxygen in a comparable patient cohort [22]. Thirteen normoxemic COPD patients with hyperinflation (mean RV 187% pred.) and EID performed ramp-incremental cycle ergometry with supplemental oxygen and NIV (16 cm H_2_O inspiratory pressure support (IPS) and 5 cm H_2_O positive end-expiratory pressure (PEEP)) or sham NIV (7 cm H_2_O IPS and 5 cm H_2_O PEEP). Improvements in central hemodynamics were reported, but no significant differences in peak work rate and ventilation were seen. Although speculative, the different ‘’sham’’ NIV, with fairly higher pressures than we used, possibly preventing small airways closure by counterbalancing the PEEPi might be one of the reasons for the difference in results [33]. Next to different settings, we also used walking on a treadmill at a constant work rate to test exercise capacity, which can be considered as endurance exercise rather than incremental exercise.

In patients with resting lung hyperinflation, the low inspiratory reserve capacity limits the possibility of Vt expansion during exercise, and thus, the maximal achievable ventilation [34,35]. Theoretically, by increasing the inspiratory capacity during exercise by providing BiPAP, the moment that patients reach their plateau in tidal volume/peak ventilation relation will be postponed. The lack of difference in isotime and end of exercise Vt and ventilation in the current study suggests all patients walked until they reached maximum ventilation, but when NIV was added to oxygen, they were able to walk longer with the same level of ventilation. These findings might imply that in our population, NIV in addition to oxygen had a more beneficial effect on dyspnea sensation and redistribution of blood flow from respiratory muscles to limb muscles rather than on respiratory mechanics. This blood flow redistribution might explain why patients experienced less leg fatigue after they received NIV and oxygen during recovery but had walked with sham settings.

Another explanation for the beneficial effect of NIV in addition to oxygen might be the improvement of the ventilation/perfusion ratio. Imaging analysis techniques have shown improvement of ventilation/perfusion and gas exchange related to long-term NIV treatment in patients with COPD [36]. However, no differences in SpO_2_ after exercise were noted when NIV was added to oxygen. In summary, the mechanisms by which NIV in addition to oxygen supplementation improved exercise endurance time in this study remain unclear.

Although overall, there are clinical significant differences in distance and recovery time, we noted heterogeneity in response among individual subjects (Figure 4), similar to other studies [14]. We used fixed ventilator settings in all patients. Individualized settings for NIV would possibly have resulted in more comparable effects for all patients. On the contrary, it seems that some patients respond better to oxygen; others respond better to NIV. This variability in responses is probably related to the differential pathological mechanisms causing patients to terminate their exercise; in some patients, this can be due to ventilatory mechanics, and in other patients, diffusion might be the dominating problem, but also variable beneficial effects on hemodynamics might play a role in individual variance. The current sample size, however, does not allow to investigate these differences further.

Recovery time is shorter with NIV and oxygen support but does not result in a longer walking distance after recovery without continuous NIV and oxygen support. It might indicate that the recovery time was sufficient to recover from the given exercise, but we did not assess muscle recovery. Indeed, prolonged skeletal muscle recovery after exercise has been observed in hypoxemic COPD patients [37]. The efficacy of postexercise O_2_ supplementation, however, is still debated [38]. Interestingly, leg fatigue was lower when subjects walked with sham conditions, but recovered with NIV and oxygen. This might be partly related to the fact that patients stopped because of dyspnea before the legs got tired. The degree of leg fatigue was indeed lower after the two settings in which patients walked with sham (i.e., ‘’sham’’ and ‘’intermittent’’ setting), although not significantly different. Conversely, leg fatigue improved more pronounced if NIV and oxygen was applied during recovery, suggesting a beneficial effect on lower limb skeletal muscle.

The isotime data did not convincingly explain the beneficial effect of NIV and supplemental oxygen compared to the other settings. However, these curves only provide data of part of the tests with oxygen with or without NIV, as the shortest (sham or intermittent) test is used to define isotime. A recently published study investigated the differences in exercise duration in 20 severely hyperinflated patients with chronic hypercapnic respiratory failure when using oxygen with or without high-pressure NIV [11]. Similar to our study, cycle endurance time was significantly longer when NIV was added to oxygen. Contrary to our study, isotime evaluation during exercise showed lower P_a_CO_2_ and higher SpO_2_ during NIV use, which may be related to different characteristics of study populations.

The current results provide evidence for individualizing pulmonary rehabilitation programs. In selected patients with high dyspnea sensation related to (dynamic) hyperinflation who are unable to perform exercise training, NIV is a viable option. It, however, remains to be studied whether and to what extent training with NIV and oxygen in this subgroup extents functional outcomes.

Strengths of this study are the standardized and highly controlled laboratory conditions, the inclusion of a sham setting and blinding of the patient for the setting used. The study also has limitations. Although, theoretically, CPAP is not a real ventilation mode, it might reduce dyspnea and increase exercise tolerance in COPD patients by reducing the DH-associated increased inspiratory threshold load on the inspiratory muscles through counterbalancing PEEPi [39,40]. The use of CPAP might also improve ventilation/perfusion in patients with COPD and hyperinflation Nevertheless, this ‘’sham’’ setting was provided to assure that patients remained blinded to the intervention being applied, and the pressure of 2 cm H_2_O should be enough to overcome the pressure gradients in the tube and mask. Second, three patients (19%) dropped out because of NIV-intolerance. This rate is comparable to other NIV studies [12,15]. Third, although the different tests were done in a consecutive clockwise rotation scheme with a different starting test setting, no real randomization was done. Because this study was not randomized, we used GEE for all available data to correct for this (data not presented), which did not influence the results. Fourth, we limited the exercise time with a maximum of six minutes for the treadmill tests, which potentially could influence exercise time interpretation. Of the 104 exercise bouts performed in total, only four of them reached the six minutes limit. Hence, we believe this effect is negligible. Given that one of these bouts was in the oxygen setting and three in the NIV/oxygen setting, the influence would be towards an underestimation of the intervention effect. Fifth, we did not study the effects of NIV without oxygen supplementation in our cohort, and therefore, we cannot draw conclusions related to an NIV only effect. Sixth, a resting V_t_ of 800 ml is relatively high, especially in COPD patients (Table 2). The V_t_ we documented is measured by the ventilator. However, non-invasive ventilators do not measure tidal volume directly but give an estimation through a built-in software [41]. We mainly used the V_t_ to evaluate the different test settings. Lastly, we studied a small sample size, and larger studies regarding this topic are needed to establish the effects of NIV in addition to oxygen in this population.

## 5. Conclusions

COPD patients with severe hyperinflation and exercise-induced desaturation benefit NIV in addition to oxygen during exercise; they walk further with the same level of dyspnea and recover faster. These results may contribute to individualized treatment during exercise training.

## Figures and Tables

**Table 1:** Baseline characteristics *n* = 13.

Baseline Characteristics	Mean
Age, years	61.5 ± 6.8
Female, *n* (%)	9 (69)
BMI, kg/m^2^	25.4 ± 5.0
mMRC score, median (IQR)	3.0 (2.0)
Ex-smoker, *n* (%)	13 (100%)
Packyears	40.8 ± 15.2
FEV1, % predicted	34.0 ± 10.2
FEV1/FVC, %	27.9 ± 4.2
FRC, % predicted	171.9 ± 18.3
RV, % predicted	193.4 ± 32.0
TLC, % predicted	128.4 ± 13.5
RV/TLC ratio	0.59 ± 0.08
DLCO, % predicted (*n* = 12)	46.7 ± 12.8
P_a_O_2_, kPa	9.2 ± 1.5
P_a_CO_2_, kPa	5.6 ± 0.6
6MWD, m	403.2 ± 73.6
6MWD, % predicted	66.1 ± 13.2
6MWD SpO_2_ start, %	92.2 ± 3.2
6MWD SpO_2_ stop, %	82.5 ± 5.1
6MWD HR start, b/min	83.0 ± 13.6
6MWD HR stop, b/min	108.2 ± 13.1
6MWD Borg D start	2.3 ± 1.3
6MWD Borg D stop	5.4 ± 1.8
6MWD Borg F start	2.2 ± 1.4
6MWD Borg F stop	4.5 ± 1.1

Abbreviations: FEV1 = forced expiratory volume in 1 second, FVC = forced vital capacity, FRC = functional residual capacity, RV = residual volume, TLC = total lung capacity, DLCO = diffusion capacity of the lung for carbon monoxide, BMI = body mass index, mMRC = modified medical research council, P_a_O_2_/P_a_CO_2_ = partial arterial pressure of oxygen/carbon dioxide, 6MWD = 6 minute walking distance, SpO_2_ = saturation of peripheral oxygen, HR = heart rate, Borg D = Borg dyspnea, Borg F = Borg fatigue.

**Table 2:** Results of 13 COPD patients with hyperinflation and exertional oxygen desaturation completing four sets of treadmill tests with different settings of the ventilator.

	Exercise Bout One				Exercise Bout Two			
	1 Sham	2 Oxygen	3 NIV and Oxygen	4 Intermittent	1 Sham	2 Oxygen	3 NIV and Oxygen	4 Intermittent
Total distance walked, m	120 (50)	225 (107) *	283 (128) ˟ ±	123 (38) • ÷	107 (69)	188 (109) *	261 (136) ˟ ±	119 (45) • ÷
Endurance time, seconds	91 (36)	171 (88) *	216 (105) ˟ ±	91 (27) • ÷	80 (47)	145 (92) *	198 (103) ˟ ±	89 (34) • ÷
Recovery time, seconds	170 (49)	150 (48)	117 (51) ˟	119 (37) º •				
Pre-test HR, bpm	84 (12.2)	84 (14.4)	83 (14.8)	84 (13.2)	91 (15.4)	91 (14.3)	92 (16.1)	89 (13.0)
Post- test HR, bpm	100 (19.9)	105 (16.7)	105 (19)	104 (15.5)	106 (13.8)	107 (17.6)	110 (17.5)	102 (13.6)
Recovery HR, bpm	91 (15.4)	91 (14.3)	92 (16.1)	89 (13.0)	92 (10.6)	92 (16.0)	94 (16.9)	93 (15.1)
Pre-test BF, bpm	21 (5.0)	21 (6.5)	18 (4.1)	20 (4.7) ÷	21 (8.0)	24 (5.6)	24 (4.3)	23 (4.5)
Post-test BF, bpm	31 (10)	28 (7.8)	27 (6.7)	32 (5.4) ÷	30 (7.7)	29 (8.1)	28 (5.8)	32 (8.7)
Recovery BF, bpm	21 (8.0)	24 (5.6)	24 (4.3)	23 (4.5)	22 (5.2)	23 (5.7)	22 (5.5)	24 (4.5)
Pre-test SpO_2_, %	94 (3.0)	96 (2.5)	98 (2.6)	95 (2.1)	93 (4.4)	98 (2.7) *	99 (1.0) ˟	99 (1.0) º
Post-test SPO_2_, %	84 (6.6)	96 (5.2) *	98 (1.2) ˟	85 (4.6) •	84 (7.0)	97 (3.2) *	99 (1.5) º	85 (4.6) • ÷
Recovery SpO_2_, %	93 (4.4)	98 (2.7) *	99 (1.0) ˟	99 (1.0) º	90 (5.0)	98 (2.6) *	99 (0.8) ˟	90 (5.9) • ÷
Pre-test tcP_a_CO_2_, kPa (*n* = 1)	5.3 (0.7)	5.4 (0.7)	5.1 (0.9)	5.2 (0.7)	6.1 (0.9)	6.7 (0.9) *	6.3 (1.2)	5.9 (0.7) •
Post-test tcP_a_CO_2_, kPa (*n* = 11)	5.6 (0.8)	6.3 (1.1) *	5.9 (1.1)	5.5 (0.7) •	6.1 (0.9)	6.6 (1.4)	6.5 (1.3)	6.0 (0.9)
Δ tcP_a_CO_2_, kPa (*n* = 11)	0.3 (0.2)	1.0 (0.9) *	0.9 (0.6) ˟	0.3 (0.3) ÷	6.4 (0.9)	7.1 (1.2) *	6.6 (1.2)	6.4 (0.9) •
Recovery tcP_a_CO_2_, kPa (*n* = 11)	6.1 (0.9)	6.7 (0.9) *	6.3 (1.2)	5.9 (0.7) •	0.02 (0.1)	-0.06 (0.8)	0.1 (0.3)	0.1 (0.2)
Pre-test tidal volume, mL (Median (IQR))	808 (354) (696 (398))	822 (383) (699 (479))	954 (214) (919 (242))	800 (298) (709 (255))	1059 (276) (1031 (405))	921 (290) * (849 (463))	1130 (284) ± (1075 (424))	1020 (245) (954 (252))
Post-test tidal volume, mL (Median (IQR))	1075 (296) (1034 (336))	1145(338) (1051 (519))	1166 (257) (1263 (441))	1119 (249) (1066 (407))	1128 (291) (1018 (381))	1122 (304) (1083 (440))	1141 (410) (1161 (463))	1039 (289) (977 (412))
Δ tidal volume, mL (Median (IQR))	267 (241) (360 (448))	323 (336) (306 (530))	212 (256) (166 (321))	318 (228) (313 (260))	68 (170) (117 (257))	201 (234) (166 (302))	11 (287) (29 (207))	19 (165) (−18 (229))
Recovery tidal volume, mL (Median (IQR))	1059 (276) (1031 (405))	921 (290) * (849 (463))	1130 (284) ± (1075 (424))	1020 (245) (954 (252))	1208 (421) (1143 (588))	1124 (437) (981 (862))	1285 (348) (1223 (357))	1202 (334) (1169 (515))
Pre-test ventilation, L/min	15.9 (3.2)	15.4 (3.1)	16.8 (4.0)	15.3 (2.6)	22.0 (8.7)	20.7 (4.7)	26.5 (5.7) ±	22.6 (5.5) ÷
Post-test ventilation, L/min	31.1 (9.8)	30.6 (8.4)	30.9 (9.3)	35.9 (10.3)	32.1 (8.0)	30.9 (8.2)	32.3 (12.2)	31.7 (8.2)
Pre-test Borg-D, points (Median (IQR))	0.7 (1.0) (0.5 (1.5))	0.8 (0.9) (0.5 (2.0))	0.5 (0.6) (0.5 (1.0))	0.8 (1.2) (0.0 (1.5))	1.8 (0.6) (2.0 (0.0))	1.9 (0.4) (2.0 (0.0))	1.8 (0.4) (2.0 (0.0))	1.7 (0.7) (2.0 (0.0))
Post-test Borg-D, points (Median (IQR))	4.1 (2.1) (5.0 (2.0))	5.2 (2.1) (4.0 (3.0))	4.3 (1.4) ± (4.0 (1.0))	5.2 (1.4) (5.0 (3.0))	4.9 (2.4) (4.0 (3.0))	4.8 (2.2) (4.0 (3.5))	4.7 (1.8) (4.0 (2.5))	5.1 (1.7) (5.0 (2.0))
Recovery Borg-D, points (Median (IQR))	1.8 (0.6) (2.0 (0.0))	1.9 (0.4) (2.0 (0.0))	1.8 (0.4) (2.0 (0.0))	1.7 (0.7) (2.0 (0.0))	1.9 (0.6) (3.0 (0.5))	1.8 (0.5) (2.0 (0.0))	1.8 (0.6) (2.0 (0.5))	2.0 (0.4) (2.0 (0.5))
Pre-test Borg-F, points (Median (IQR))	1.0 (1.1) (0.5 (2.0))	1.0 (1.1) (0.25 (2.0))	0.6 (0.6) (0.75 (1.0))	0.6 (0.8) (0.25 (1.0))	1.7 (0.9) (2.0 (1.0))	1.8 (1.0) (2.0 (2.0))	1.7 (1.2) (1.0 (2.0))	0.6 (0.8) º • ÷ (0.0 (1.0))
Post-test Borg-F, points (Median (IQR))	2.8 (1.9) (2.0 (2.5))	3.3 (1.6) (3.0 (1.0))	3.1 (1.5) (3.0 (2.0))	2.6 (2.0) (3.0 (2.8))	2.4 (1.4) (2.0 (2.5))	2.8 (1.3) (3.0 (1.0))	3.2 (1.9) (3.0 (2.5))	2.3 (1.6) (2.0 (2.3))
Recovery Borg-F, points (Median (IQR))	1.7 (0.9) (2.0 (1.0))	1.8 (1.0)(2.0 (2.0))	1.7 (1.2) (1.0 (2.0))	0.6 (0.8) º • ÷ (0.0 (1.0))	1.9 (0.8) (2.0 (1.0))	1.7 (1.1) (2.0 (2.0))	1.8 (1.0) (1.0 (0.5))	1.6 (0.8) (1.0 (1.0))

Statistical significant differences per bout of exercise (*p* < 0,05); * = 1 vs. 2, ˟ = 1 vs. 3, º = 1 vs. 4, ± = 2 vs. 3, • = 2 vs. 4, ÷ = 3 vs. 4; Abbreviations: Δ = delta, Borg D = Borg dyspnea, Borg F = Borg fatigue, SpO_2_ = saturation of peripheral oxygen, HR = heart rate, BF = breathing frequency, tcP_a_CO_2_ = transcutaneous partial pressure of carbon dioxide, V_T_ = tidal volume, IQR = interquartile range.
